# Thymoquinone Inhibits the CXCL12-Induced Chemotaxis of Multiple Myeloma Cells and Increases Their Susceptibility to Fas-Mediated Apoptosis

**DOI:** 10.1371/journal.pone.0023741

**Published:** 2011-09-01

**Authors:** Gamal Badr, Eric A. Lefevre, Mohamed Mohany

**Affiliations:** 1 Zoology Department, College of Science, King Saud University, Riyadh, Saudi Arabia; 2 Zoology Department, Faculty of Science, Assiut University, Assiut, Egypt; 3 Institute for Animal Health, High Street, Compton, Newbury, United Kingdom; Wayne State University, United States of America

## Abstract

In multiple myeloma (MM), malignant plasma cells reside in the bone marrow, where they accumulate in close contact with stromal cells. The mechanisms responsible for the chemotaxis of malignant plasma cells are still poorly understood. Thus, we investigated the mechanisms involved in the chemotaxis of MDN and XG2 MM cell lines. Both cell lines strongly expressed CCR9, CXCR3 and CXCR4 chemokine receptors but only migrated toward CXCL12. Activation of CXCR4 by CXCL12 resulted in the association of CXCR4 with CD45 and activation of PLCβ3, AKT, RhoA, IκBα and ERK1/2. Using siRNA-silencing techniques, we showed CD45/CXCR4 association is essential for CXCL12-induced migration of MM cells. Thymoquinone (TQ), the major active component of the medicinal herb Nigella sativa Linn, has been described as a chemopreventive and chemotherapeutic compound. TQ treatment strongly inhibited CXCL12-mediated chemotaxis in MM cell lines as well as primary cells isolated from MM patients, but not normal PBMCs. Moreover, TQ significantly down-regulated CXCR4 expression and CXCL12-mediated CXCR4/CD45 association in MM cells. Finally, TQ also induced the relocalization of cytoplasmic Fas/CD95 to the membrane of MM cells and increased CD95-mediated apoptosis by 80%. In conclusion, we demonstrate the potent anti-myeloma activity of TQ, providing a rationale for further clinical evaluation.

## Introduction

Multiple myeloma (MM) is a malignant plasma cell disorder that accounts for approximately 10% of all hematologic cancers [1] and is the second most prevalent hematologic malignancy. Although some therapeutic drugs and novel therapies are available, MM remains incurable, and the median survival time for MM patients is 3 to 5 years [2], [3]. Several chemotherapeutic agents (e.g., vincristine, dexamethasone and melphalan) are currently used to treat MM. However, these drugs increase the risk of developing secondary hematologic malignancies such as therapy-related myelodysplatic syndromes [4]. Therefore, there is a need to further identify the factors and mechanisms that are responsible for maintaining the survival of MM cells and mediating tumorigenesis and drug resistance. Although the presence of circulating malignant plasma cells has been demonstrated in more than 70% of patients diagnosed with MM [5], the survival of MM cells requires their migration from the blood to the bone marrow (BM) niches (known as homing) and active navigation from one area to another within the BM [6]. Several studies have revealed the important role of chemokines and their receptors in the chemotaxis and pathogenesis of MM cells [7], [8]. MM cells express variable levels of the chemokine receptors CXCR3, CXCR4, CCR1, CCR5, and CCR6 and show variable responses to their cognate ligands CCL3, CXCL12 and CCL5 [9], [10]. In addition, Vande Broek *et al.* reported the involvement of CCR2 and its ligands CCL2, CCL8 and CCL7 in the migration of human MM cells towards the BM [11].

Of the numerous chemokine receptors that are expressed, CXCR4 is the most highly expressed chemokine receptor in many cancer cells [12]. CXCR4 ligand, CXCL12, is strongly expressed in lung, liver, bone marrow and lymph nodes, which are all common metastatic destinations for many types of cancer. Moreover, upregulation of CXCR4 has frequently been observed in various cancers, including colon carcinoma, lymphoma, breast cancer, glioblastoma, leukemia, prostate cancer, oral squamous cell carcinoma and pancreatic cancer [9], [13], [14], [15], [16], [17], [18]. Similarly, several studies have shown that CXCR4 is also the most abundant and functional of the chemokine receptors expressed by MM cells, and thus may play a major role in the pathogenesis of the disease. Indeed, published data suggest the involvement of CXCL12/CXCR4 in the maintenance and survival of MM cells in both *in vivo* and *in vitro* models [19], [20]. Nevertheless, the signaling pathways activated downstream of CXCR4 after stimulation with CXCL12 in MM cells remain poorly defined.

Recently, natural antioxidants have been used as adjuvants in combination with chemotherapy to reduce the side effects and increase the efficiency of cancer treatments [21]. In particular, antioxidants are very effective at inducing the growth arrest of human colon cancer cells [22]. In recent years, numerous natural products have been evaluated for their use in cancer treatment [23], [24].

Thymoquinone (TQ), the most abundant component of black seed oil extract, is the active compound that is primarily responsible for many of the seed's antioxidant and anti-inflammatory effects [25]. TQ exerts an anti-neoplastic effect and may be a promising dietary chemopreventive agent. In particular, several studies have illustrated the pro-apoptotic and growth arrest effects of TQ on several cancer cell lines [26]. In addition, TQ exerts inhibitory effects on the cell proliferation in many types of cancer cells, including breast and ovarian adenocarcinoma [27], colorectal cancer [28], human pancreatic adenocarcinoma and uterine sarcoma [25], neoplastic keratinocytes [29], human osteosarcoma [30] and fibrosarcoma and lung carcinoma [31]. An *in vivo* study by Badary and El-Din showed that TQ-treatment inhibited a fibrosarcoma tumor incidence and burden by 43 and 34%, respectively [32]. Recently, Ravindran et al. published a detailed study of the anti-proliferative, anti-inflammatory and chemosensitization activities of TQ in myeloid leukemia cells [33]. In this study, they also compared the effect of TQ on various cancer cells, and showed that TQ exerts an anti-proliferative effect on MM (U266) cells, although the authors did not address the underlying mechanisms.

In the present study, we investigated the chemokines responsible for and the precise mechanisms involved in MM cell chemotaxis. Although TQ is known to modulate the proliferation of and induce growth arrest in numerous cancer cells, very few data are available concerning its effect on MM cells. For this reason, we also evaluated the efficiency of TQ in modulating the chemotaxis and survival of MM cells.

## Materials and Methods

### Cells and reagents

IL-6–dependent human myeloma MDN and XG-2 cell lines [34], [35] were obtained from the Veterinary Serum and Vaccine Research Institute (VSVRI, Cairo, Egypt). Both cell lines were maintained in RPMI 1640 containing 10 mM HEPES, 10% fetal calf serum (FCS; Biowittaker, Walkersville, MD) and 1% L-glutamate (R-10 medium) supplemented with 3 ng/mL IL-6 (Peprotech, Rocky Hill, NJ). The cell cultures were free of *Mycoplasma*, as assessed by an enzyme-linked immunosorbent assay (ELISA) kit (Boehringer, Mannheim, Germany).

BM aspirate samples obtained from 9 patients with MM were kindly provided by Dr. Douaa Sayed (South Egypt Cancer Institute, Assiut University). Plasma cells were isolated from the BM aspirates by positive selection using anti-CD138 antibody-coated immunomagnetic beads according to the manufacturer's instructions (MACS, Miltenyi Biotech). MACS purification consistently resulted in a >95% pure primary MM cell population (as determined by monitoring cell-surface expression of CD38 and CD45) and a viability of >90% (as determined using the trypan blue exclusion method). Peripheral blood mononuclear cells (PBMCs) from healthy donors were purified using a standard Ficoll-Paque gradient centrifugation method according to the instructions of the manufacturer (Amersham Pharmacia, Uppsala, Sweden). Briefly, the heparinized blood was diluted 1∶1 in phosphate-buffered saline (PBS), carefully layered over the Ficoll-Paque gradient, and centrifuged for 20 min at 1,020× *g*. The cell interface layer was carefully harvested, and the cells were washed twice in PBS and resuspended in RPMI 1640. In some experiments, cells were pre-treated using one or a combination of the following compounds (unless otherwise stated, all compounds were obtained from Calbiochem, San Diego, CA): (i) 10 µg/ml brefeldin A (Sigma-Aldrich) or 1 µM cycloheximide (Sigma-Aldrich) at 37°C for 30 min; (ii) TQ (Sigma-Aldrich, UK) at 37°C for various time lengths (as indicated in the figure legends); and (iii) 5 µg/ml AMD3100 (AMD, a CXCR4 antagonist), 100 nM or 1 µM wortmannin (WM, a phosphatidylinositol-3 kinase [PI3K] inhibitor that inhibits all classes of PI3K at 1 µM but is selective for class I PI3K at 100 nM), 10 µM PD98059 (a mitogen activated protein kinase [MEK1/2] inhibitor), 10 µM SB203580 (a P38 MAPK inhibitor), 100 nM U73122 (a phospholipase C [PLC] inhibitor) or its inactive control U73343, 1 µM SN50 (an inhibitor of NF-κB nuclear translocation), 50 µg/ml Y27632 (a RhoA inhibitor), 100 ng/ml pertussis toxin (PTX, an inhibitor of heterotrimeric G-protein signaling), 5 µM SH5 (a PDK1 inhibitor, Alexis, Coger France), Na_3_VO_4_ (a protein tyrosine phosphatase inhibitor, Sigma-Aldrich) or DMSO at 37°C for one hour.

### Ethics statement

BM aspirate samples were obtained from patients at Assiut University Hospital. All aspects of this study were approved by Assiut University's ethical committee, and all patients provided a written informed consent accordingly to the Declaration of Helsinki.

### Small interfering RNA (siRNA)-mediated knockdown

SiRNA-mediated knockdown of CD45 was performed using synthetic duplex RNA oligonucleotides as previously described [36]. The 19-nucleotide target for the CD45 and control siRNAs was GUCUUUGUCACAGGGCAAA (Dharmacon, Inc., Boulder, CO). MM cells were transfected with 100 nM siRNA by electroporation using an Amaxa system (Amaxa GmbH, Cologne, Germany), and CD45 siRNA-mediated knockdown was confirmed after 48 h by flow cytometry.

### Flow cytometry

Cell surface antigen expression was determined by single-parameter fluorescence-activated cell sorter (FACS) analysis using the following monoclonal antibodies (mAbs): (i) PE-conjugated anti-CCR1, anti-CCR3, anti-CCR5 (clone 45531.111), anti-CCR7 (clone 150503), anti-CCR6 (clone 53103.111), anti-CXCR3, anti-CXCR4 (clone 44717.111) and anti-CXCR5, all purchased from R&D Systems; and (ii) PE-conjugated anti-CD38, anti-CD138, anti-CD45 and anti-CD95 (IgG1) mAbs, FITC-conjugated anti-CD62L, anti-VLA4, anti-α4β1, anti-α4β7 and anti-CD54 mAbs, and FITC- and PE-conjugated mouse isotype-matched control mAbs, all purchased from BD Biosciences. To detect the expression of cytoplasmic CD95, MM cells were permeabilized using a Cytofix/Cytoperm kit (BD Biosciences) according to the manufacturer's instructions before being stained with PE-conjugated anti-CD95 mAb or mouse IgG1. A FACScan™ flow cytometer with CellQuest® software (BD Biosciences) was used for data acquisition and analysis. After gating on viable cells, 15,000 events per sample were analyzed. For each marker, the threshold of positivity was defined beyond the nonspecific binding observed in the presence of a relevant isotype control mAb.

### *In vitro* chemotaxis assays

The chemokine-dependent migration of MM cells and PBMCs was measured using an in vitro 2-chamber migration assay (using Transwell plates purchased from Costar, Cambridge, MA) followed by flow cytometry analysis. All chemotaxis assays were performed in pre-warmed migration buffer (RPMI 1640 containing 1% FCS). A total of 600 µl of migration buffer alone or supplemented with either CCL3, CCL4, CCL5, CCL25, CXCL9, CXCL10 (all at 500 ng/ml) or CXCL12 (at 250 ng/ml) (all from R&D Systems) was added to the lower chamber and 10^5^ cells in migration buffer were added to the upper chamber. The plates were then incubated for 3 h at 37°C, and input cells and transmigrated cells were centrifuged, fixed in 300 µl of 1× PBS+1% formaldehyde and counted for 60 seconds using a FACscan™ flow cytometer (Beckton Dickinson). The percentage of migration was calculated as the percentage of input cells that migrated to the lower chamber. To calculate the percentage of specific migration induced by chemokines, the percentage of cells migrating to medium alone was subtracted from the percentage of cells migrating to medium with chemokines.

To test the effect of various inhibitors on chemotaxis, cells were pre-treated as detailed in the “cells and reagent” section before their addition to the upper chamber. The percentage of inhibition of specific migration in the presence of inhibitors was calculated as follows: [1 - (specific migration of treated cells/specific migration of untreated cells)]×100.

### Western blots

Untreated and TQ-treated myeloma cells (5×10^6^ cells/ml in pre-warmed R-10 medium without FCS) were stimulated for 2 min at 37°C with or without 250 ng/ml CXCL12. Lysates were prepared as previously described [37]. Equal amounts of total cellular protein were resolved using SDS-polyacrylamide gel electrophoresis (SDS-PAGE) and analyzed by western blotting. Antibodies recognizing phospho-PKB/AKT (S473), PKB/AKT, phospho-ERK1/2 (T202/Y204), phospho-IκBα (S32/36), IκBα, phospho-PLCβ3 (S537), PLCβ3, phospho-P38 MAPK (T180/Y182), P38 MAPK, and pan CD45 (all from New England Biolabs, Beverly, MA) or ERK1/2 (Santa Cruz Biotechnology, Santa Cruz, CA) were used in combination with horseradish peroxidase-conjugated secondary antibodies. Protein bands were detected using enhanced chemiluminescence reagents (ECL, Supersignal Westpico chemilumiscent substrate, Perbio, Bezons, France), and the ECL signal was recorded on hyperfilm ECL. To quantify band intensities, films were scanned, saved as TIFF files and analyzed using NIH Image J software.

For immunoprecipitation, cell lysates containing equal amounts of protein were clarified by incubation with Protein A-Sepharose CL-4B or GammaBindTM-Sepharose beads (Amersham Biosciences) for 1 h at 4°C. The Sepharose beads were removed by brief centrifugation, and the supernatants were incubated with specific primary antibodies for 2 h at 4°C. Subsequently, immunoprecipitation of the antigen/antibody complexes was performed by overnight incubation at 4°C with 50 µl of Protein A-Sepharose or GammaBindTM-Sepharose (50% suspension). Nonspecific proteins were removed by washing the beads three times with PBS. Immunoprecipitated complexes were solubilized in 50 µl of 2× Laemmli buffer and further analyzed by western blotting as described above. In some experiments, MDN and XG2 cells were incubated with or without 2 mM methyl-beta-cyclodextrin (MβCD) for 30 min or 10 µM TQ for 1 h prior to the 2 min incubation with or without 250 ng/ml CXCL12 and subsequent cell lysis.

To assess RhoA activation, cells (5×10^6^ per condition) were starved for 2 h in pre-warmed R-10 medium without FCS, incubated for 2 min at 37°C with or without 250 ng/ml CXCL12, and lysed as described above (using 200 µl of lysis buffer). After centrifugation, a 15 µl aliquot of the supernatant was kept as total lysate sample. The remaining supernatant (185 µl) was incubated for 16 h at 4°C with GST-C21 [38] precoupled to glutathione–agarose beads (Sigma, France). Beads were washed using an excess of lysis buffer and the active RhoA (RhoA_GTP_ pulled down from the lysate and bound to the fusion protein/beads) was eluted using Laemli sample buffer. Analysis by SDS-PAGE and western blotting using an anti-RhoA mAb (Santa Cruz Biotechnology, Santa Cruz, CA) was subsequently performed on both the total cell lysates and the samples eluted in Laemli buffer to detect total RhoA and RhoA_GTP_, respectively.

### Apoptosis Assays

To assess CD95-mediated apoptosis, untreated and TQ-treated myeloma cells (10^6^ cells/ml) were incubated for 30 min with or without a general caspase inhibitor (Caspase inhibitor II; Calbiochem, San Diego, CA), prior to incubation for 24 h at 37°C with 1 µg/ml B-G27 (IgG2a, Diaclone SA, Besançon, France) or mouse IgG2a as a control. Myeloma cells were then collected, and the percentage of cells undergoing apoptosis was determined as described below.

Cells were washed and incubated in PBS containing 30% human AB serum at 4°C for 30 min prior to staining with Annexin V-FITC and PI for 15 min at 25°C using a commercial kit according to the manufacturer's instructions (ABCam, Canada). Cells were analyzed by flow cytometry within 1 hour of staining, and apoptotic cells were determined as PI^neg^/Annexin V^pos^, dead cells as double positive and viable cells as double negative.

### Statistical analysis

Data were analyzed using SPSS software version 16 and are expressed as means ± SEM. Differences between groups were assessed using the analysis of variance (ANOVA) method. Data were considered significant if the calculated P values were <0.05.

## Results

### CXCL12-mediated MM cell chemotaxis involves PLC, PI3K, RhoA, NF-κB and ERK1/2

**Figure 1:**
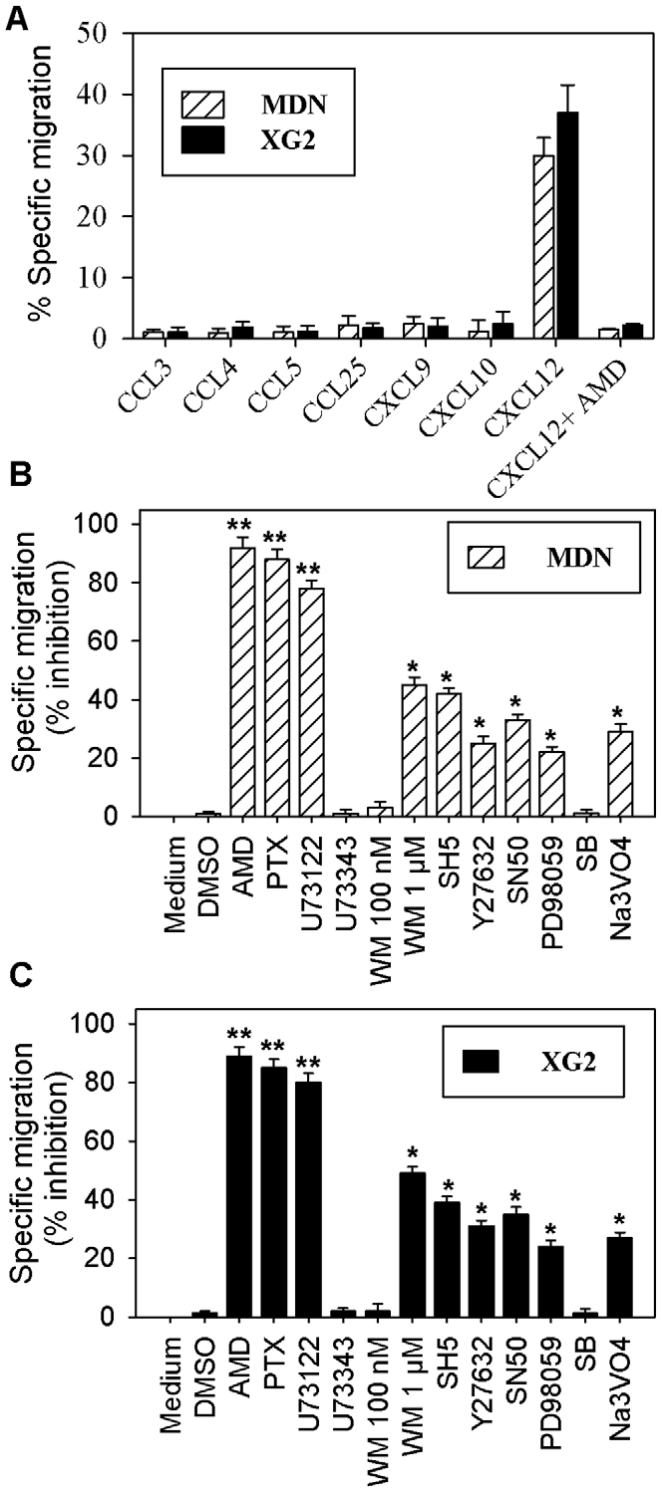
Chemotactic response of MM cells. Migratory responses of MDN and XG2 cells to the indicated chemokines were determined using Transwell plates. After incubation for 3 h at 37°C, the chemotactic response to CCL3, CCL4, CCL5, CCL25, CXCL9, CXCL10 (all at 500 ng/ml) or CXCL12 (at 250 ng/ml) was determined by flow cytometry. Some cells were pre-incubated for one hour at 37°C with AMD, before being used for the chemotaxis assay in migration medium with 250 ng/ml CXCL12. The experiment was performed in triplicate, and results are expressed as the mean percentage of specific migration ± SEM in response to each chemokine (**A**). MDN (**B**) and XG2 (**C**) cells were incubated for one hour at 37°C with medium, DMSO or various inhibitors, before being used for a chemotaxis assay in migration medium with or without 250 ng/ml CXCL12. Data from six independent experiments is expressed as the mean percentage of inhibition of specific migration ± SEM. PTX = pertussis toxin; SB = SB203580. * P<0.05and ** P<0.001.

We first investigated the surface expression of various chemokine receptors on MDN and XG2 MM cell lines using flow cytometry. Our results showed that XG2 cells strongly expressed CCR9, CXCR3 and CXCR4, expressed low levels of CCR1, CCR5, CCR7 and CXCR5, and did not express CCR6 (**Figure S1**). A similar expression pattern was observed in MDN cells (data not shown). To further analyze the functionality of the chemokine receptors expressed by MM cells, we performed a chemotaxis assay to analyze the ability of MM cells to migrate toward 500 ng/ml CCL3, CCL4, CCL5 (CCR1, CCR3 and CCR5 ligands, respectively); CCL25 (CCR9 ligand); CXCL9 or CXCL10 (CXCR3 ligand); or 250 ng/ml CXCL12 (CXCR4 ligand). Both MM cell lines only exhibited a specific migratory response to CXCL12; the percentages of cells specifically migrating to CXCL12 were 30±2.9% and 37±4.7% for MDN and XG2 cells, respectively (**Figure 1A**). The CXCL12-mediated MM cell chemotaxis was dependent on the interaction of CXCL12 with CXCR4. Indeed, the specific migration towards CXCL12 was totally inhibited in MM cells pretreated with AMD (**Figure 1A**), a specific CXCR4 antagonist that inhibits the binding and function of CXCL12 with high affinity and potency [39].

The signaling pathways involved in MM cell chemotaxis are poorly defined; therefore, we analyzed the effect of various inhibitors on CXCL12-mediated MDN and XG2 cell chemotaxis. Migration of MDN cells toward CXCL12 was strongly inhibited by AMD, PTX and U73122 (94±3.5%, 84±3.2% and 82±2.9% inhibition of migration, respectively; n = 6) and to a lesser extent by 1 µM of WM, SH5, Y27632, SN50, PD98059 and Na_3_VO_4_ (45±1.5%, 42±1.9%, 25±1.8%, 35±2.1%, 20±1.8% and 30±2% inhibition of migration, respectively; n = 6) (**Figure 1B**). Similarly, the migration of XG2 cells toward CXCL12 was also strongly inhibited by AMD, PTX and U73122 and to a lesser extent by 1 µM WM, SH5, Y27632, SN50, PD98059 and Na_3_VO_4_ (**Figure 1C**). In all experiments, the addition of DMSO, 100 nM WM, U73343 or SB203580 had no effect on the CXCL12-mediated chemotaxis of MM cells. The near total inhibition of migration toward CXCL12 observed in the presence of the CXCR4 antagonist (AMD) and the inhibitor of heterotrimeric G-proteins (PTX) illustrated the specificity of MM cell migration and confirmed that it was dependent on the interaction of CXCL12 with its receptor CXCR4 on the surface of MM cells. Importantly, the use of selective inhibitors revealed that MM cell migration toward CXCL12 was strongly dependent on PLC activity and to a lesser extent on PI3K, RhoA, NF-κB and ERK1/2 signaling. In addition, our results suggested that only class II PI3Ks were involved in MM cell migration toward CXCL12, since only the addition of 1 µM WM (which inhibits all classes of PI3K) but not 100 nM WM (which selectively inhibits Class I PI3K) impaired CXCL12-induced MM cell migration.

### CXCL12 strongly enhances PLCβ3, AKT, IκBα and ERK1/2 phosphorylation and the activation of RhoA via PI3K in MM cells

**Figure 2:**
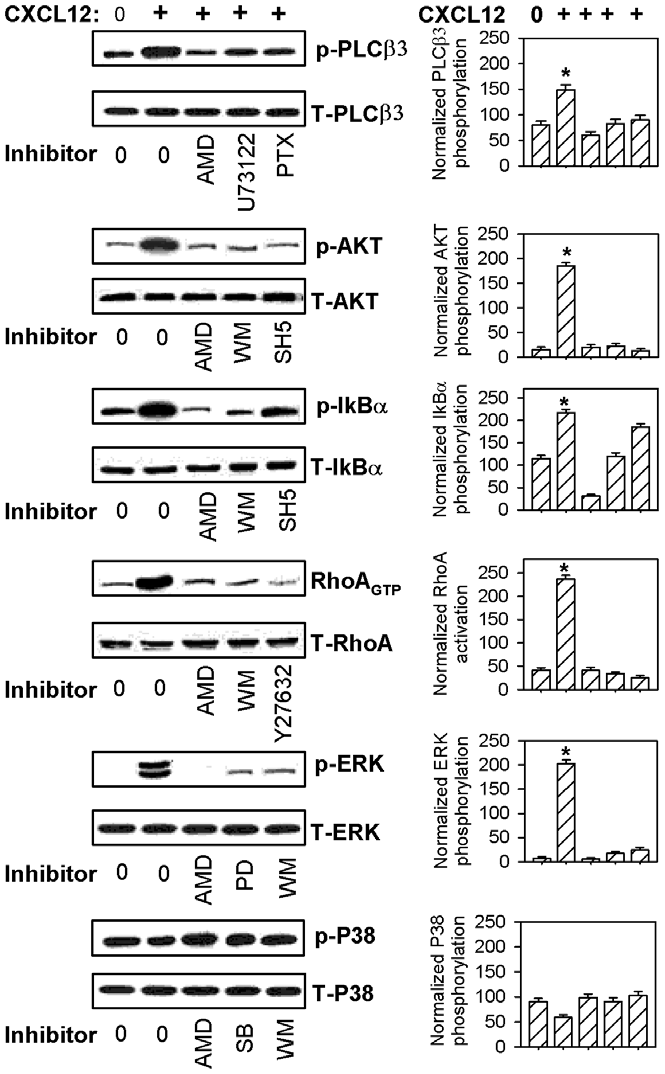
CXCL12 induces the phosphorylation of PLCβ3, AKT, ERK1/2, and IκBα and the activation of RhoA in MDN cells. MDN cells were incubated for one hour at 37°C in medium either with or without various inhibitors (WM used at 1 µM). Cells were then incubated for 2 min with medium or 250 ng/ml CXCL12 prior to being lysed. Proteins in the cell lysates were resolved on a 7% acrylamide gel. The phosphorylation levels of PLCβ3 (p-PLCβ3), ERK1/2 (p-ERK1/2), AKT (p-AKT), IκBα (p- IκBα) and P38 (p-P38) and the activation level of RhoA (RhoA_GTP_) were corrected for total relevant protein (T-PLCβ3, T-ERK1/2, T-AKT, T- IκBα, T-P38 and T-RhoA) on stripped blots. A representative blot for each downstream effector from 6 independent experiments is shown (left panel); all results are expressed as mean values of normalized specific phosphorylation ± SEM from six separate experiments (right panel). PTX = pertussis toxin; PD = PD98059; SB = SB203580. *P<0.05.

To better characterize the molecular mechanisms by which CXCL12 induces the chemotaxis of MM cells, we investigated the effect of CXCL12 on the activation of various downstream effectors of CXCR4. Since CXCL12-mediated chemotaxis of MM cells involves PLC, PI3K, RhoA, NF-κB and ERK1/2, we specifically investigated the phosphorylation status of PLCβ3, AKT, IκBα and ERK1/2 and the activation status of RhoA (an important protein for the adhesion of MM cells) following the stimulation of MDN cells with CXCL12 (**Figure 2**). In addition, P38 phosphorylation was assessed and used as a negative control, since we showed that its specific inhibitor, SB203580, did not modulate CXCL12-induced MM cell migration.

Although PLCβ3 showed some levels of phosphorylation under basal conditions, CXCL12 increased its phosphorylation level by a factor of 1.9 fold (from 80±6 to 149±9.7; n = 6). CXCL12-induced increase in PLCβ3 phosphorylation was abolished by pre-treatment with AMD (60±7.1; n = 6), U73122 (83±8.2; n = 6) or PTX (88±7.9; n = 6). In contrast, the addition of U73343, the inactive form of the PLC inhibitor U73122, did not inhibit CXCL12-induced increase in PLCβ3 phosphorylation (data not shown). To measure PI3K activity, we investigated the level of PKB/AKT phosphorylation (p-AKT) in CXCL12-stimulated MM cells. We found that CXCL12 strongly induced AKT phosphorylation (from 15±4.7 to 185±6.4; n = 6) and that this effect was abolished by pre-treatment with AMD, WM or SH5 (20±4.1, 22±5.1 and 13±3, respectively; n = 6).

Similarly to PLCβ3, we also detected a basal level of IκBα phosphorylation, which was increased by a factor of 1.7 following CXCL12 stimulation (from 128±6 to 222±8.8; n = 6). The CXCL12-induced IκBα phosphorylation was strongly inhibited by the addition of AMD and WM (31±3.6 and 120±7.7, respectively, n = 6) but only partially decreased by SH5 (185±8.3; n = 6). CXCL12 also strongly induced ERK1/2 phosphorylation (from 7±3.4 to 203±8.7; n = 6), and this effect was totally inhibited by pre-treatment with AMD, PD98059 or WM (6±2.2, 16±3.2 and 24±5, respectively; n = 6). As expected, CXCL12 did not modulate P38 phosphorylation. Our results also showed that only low levels of RhoA_GTP_ were detected in unstimulated MDN cells (42±4; n = 6) and that RhoA activation was strongly enhanced by CXCL12 stimulation (237±7.9; n = 6). The addition of AMD, WM or Y27632 abolished the CXCL12-mediated increase of RhoA_GTP_ (42±6, 34±3 and 25±4, respectively; n = 6). Similar results were obtained when using XG2 cells instead of MDN cells in these assays, and we also determined that CXCL12-induced phosphorylation of ERK1/2, IκBα and AKT peaked between 1 and 5 min in both MM cell lines (data not shown).

### CXCL12-induced MM cell chemotaxis requires the association of CXCR4 with CD45

**Figure 3:**
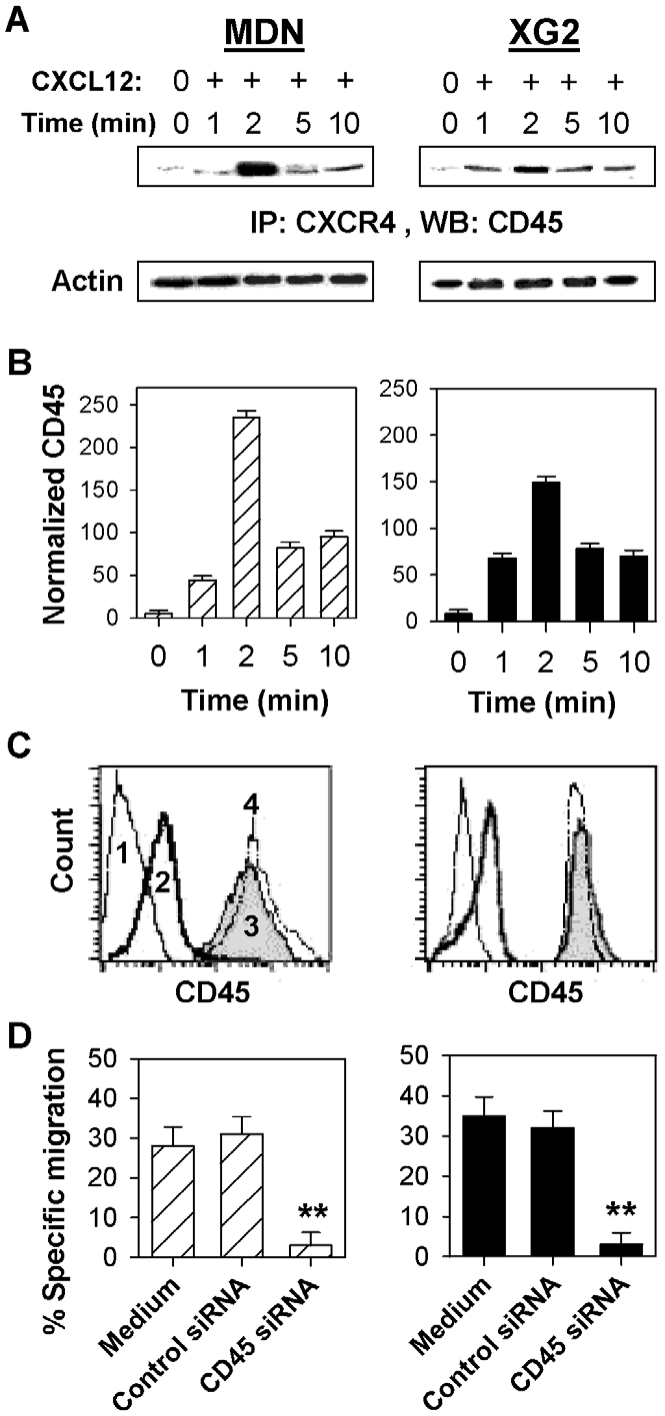
CXCL12-induced interaction of CD45 with CXCR4 is required for MM cell chemotaxis. (**A**) MDN and XG2 cells were either unstimulated (0) or stimulated with 250 ng/ml CXCL12 (+), and lysed at the indicated time points following CXCL12 stimulation (in min). Total cells lysates (equivalent to 50 µg of protein) were immunoprecipitated (*IP*) using a CXCR4 antibody. The immune complexes were resolved on a 7% acrylamide gel, transferred to a nitrocellulose membrane, and immunoblotted with an anti-CD45 antibody. Equal protein loading levels were confirmed by resolving 50 µg of total cell lysates by SDS-PAGE and immunoblotting with an anti-actin antibody. A representative blot from 6 independent experiments is shown. (**B**) Results from figure 3A are expressed as mean normalized CD45 levels ± SEM from six separate experiments. (**C**) Untransfected (gray filled histograms; 3), control siRNA-transfected (thin dotted line histograms; 4) and CD45 siRNA-transfected (bold solid line histograms; 2) MDN (left panel) and XG2 (right panel) cells were cultured for 48 hours, stained with an anti-CD45 antibody and analyzed by flow cytometry. Cells stained with an isotype-matched mAb are shown as the negative control (thin solid line histograms; 1). (**D**) Specific migration of untransfected, control siRNA- and CD45 siRNA-transfected cells was assessed in migration medium with or without 250 ng/ml CXCL12 by flow cytometry. Data is expressed as the mean percentage of specific migration ± SEM from six independent experiments for both MDN (hatched bars) and XG2 (closed black bars) cells. ** P<0.004.

Fernandis et al. previously demonstrated that the membrane tyrosine phosphatase CD45 interacts with CXCR4 and regulates the CXCR4-mediated chemotactic activity of cells from the Jurkat human T-cell line [40]. Using co-immunoprecipitation and immunoblotting techniques, we showed that CXCL12 stimulation similarly induced a physical association between CXCR4 and CD45 in both MM cell lines tested (**Figure 3A and 3B**). This association was rapid, reaching its maximum level at 2 min post-stimulation. Using siRNA silencing techniques, we successfully and specifically abrogated CD45 expression in these MM cell lines (**Figure 3C**) and showed that CD45 siRNA-transfected (but not control siRNA-transfected) MDN and XG2 cells lost their ability to migrate in response to CXCL12 (**Figure 3D**). Our results thus illustrate the critical role of CD45 expression and interaction with CXCR4 in the CXCL12-induced chemotaxis of MM cells.

### Thymoquinone inhibits the chemotaxis of MM cells in a dose- and time-dependent manner

**Figure 4:**
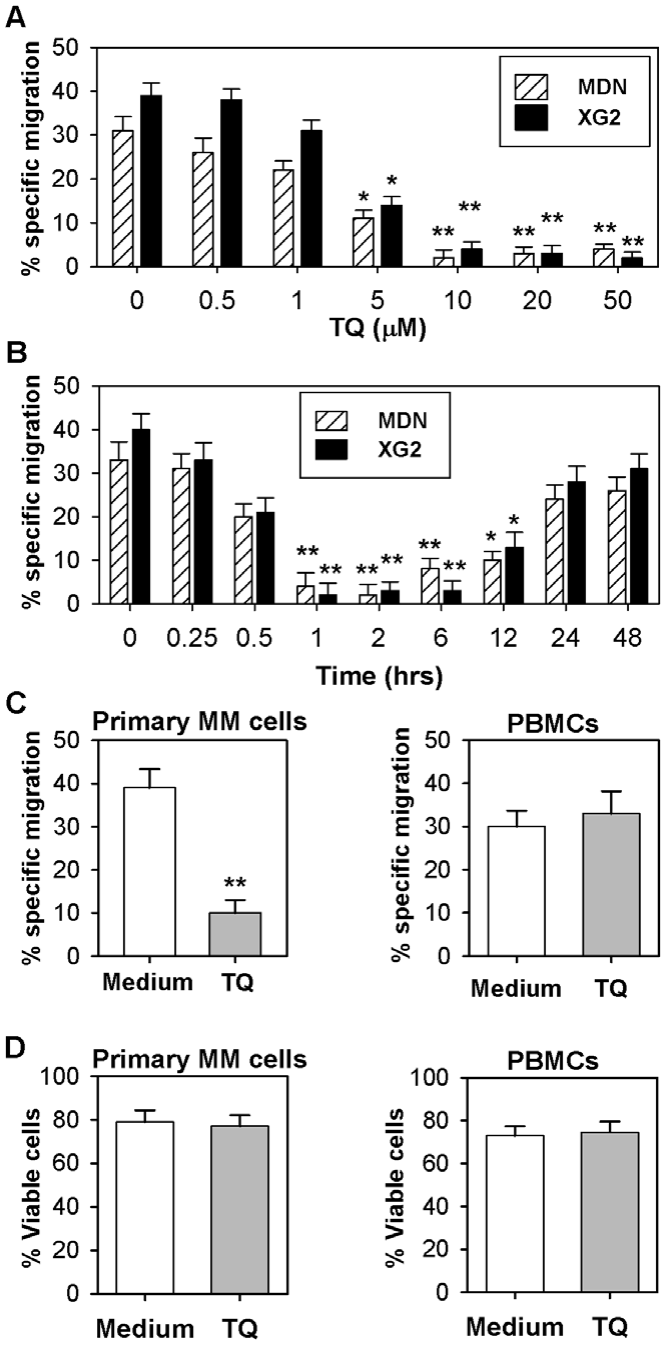
Dose- and time-dependent effects of TQ on CXCL12-mediated MM cell chemotaxis. (**A**) MDN (hatched bars) and XG2 (closed black bars) cells were incubated for 2 hours in medium with or without increasing concentrations of TQ, and their chemotactic response to 250 ng/ml CXCL12 was analyzed by flow cytometry. The experiment was performed in triplicate, and results are expressed as the mean percentage of specific migration ± SEM. * P<0.05, ** P<0.02. (**B**) MDN and XG2 cells were incubated for the indicated time periods with 10 µM TQ prior to assessing their chemotactic response to 250 ng/ml CXCL12. The experiment was performed in triplicate, and results are expressed as the mean percentage of specific migration ± SEM. * P<0.05, ** P<0.02. (**C**) Primary MM cells isolated from the BM of patients and PBMCs from healthy donors were incubated in R-10 medium for six hours prior to incubation in medium with (gray bars) or without (open bars) 10 µM TQ for one hour at 37°C. Their chemotactic response to 250 ng/ml CXCL12 was subsequently assessed by flow cytometry. The experiment was performed in triplicate with cells from nine different donors, and the results are expressed as the mean percentage of specific migration ± SEM. ** P<0.02. (**D**) Primary MM cells isolated from the BM of patients and PBMCs from healthy donors were cultured for 6 hours in migration medium before being incubated for one hour at 37°C in medium either with (hatched bars) or without (open bars) TQ. The percentage of viable cells was determined by Annexin-V staining and flow cytomety. Results are expressed as the mean percentage of viable cells ± SEM from nine separate experiments.

MDN and XG2 MM cells were cultured in the presence or absence of TQ at various concentrations, and we subsequently determined their ability to migrate in response to CXCL12. The percentages of specific migration of untreated MDN and XG2 cells to CXCL12 were 31±3.2% and 39±2.9%, respectively. Our results showed that pre-treatment with TQ strongly impaired the chemotaxis of both MDN and XG2 cells toward CXCL12. This effect was dose-dependent and peaked at 10 µM TQ; at this dose, the percentages of specific migration of TQ-treated MDN and XG2 cells toward CXCL12 were only 2% and 4%, respectively (**Figure 4A**). This inhibition of MM cell migration did not result from a TQ-mediated cytotoxic effect, since the addition of TQ (even at the highest concentration) had no effect on cell viability as assessed by the trypan blue exclusion method (data not shown). MDN and XG2 cells were then cultured in the presence of 10 µM TQ for increasing lengths of time prior to assessing their migration toward CXCL12. We found that the maximal TQ-induced inhibition of CXCL12-mediated MM cell chemotaxis was observed following a one to six hour pre-treatment with TQ. Indeed, the chemotactic response of MDN cells was decreased by 87.9% (n = 6) and the response of XG2 cells was decreased by 95% (n = 6) after pre-incubation with TQ for 1 h (**Fig. 4B**).

We then evaluated the effects of TQ on the CXCL12-induced migration of primary MM cells (purified from bone marrow aspirates of MM patients) and PBMCs isolated from healthy donors. In the absence of TQ, 39±4.3% (n = 9) of primary MM cells and 30±3.7% (n = 9) of PBMCs from healthy donors specifically migrated in response to CXCL12. Following TQ pre-treatment, MM cell migration toward CXCL12 was inhibited by 74% (from 39±4.3% to 10±3%; n = 9), whereas the migratory capacity of PBMCs from healthy donors toward CXCL12 was not affected (from 30±3.7% to 34±5.2%; n = 9) (**Fig. 4C**). Using similar culture conditions, the percentage of viable cells (Annexin-V^neg^/PI^neg^) was similar in both untreated and TQ-treated primary MM cells (80.8±3.3 and 79.5±2.6, respectively; n = 9) and PBMCs (76±3.5 versus 78±3.1, respectively; n = 9) (**Fig. 4D**). Therefore, our results showed that TQ specifically inhibited the CXCL12-induced chemotaxis of MM cells without inducing any cytotoxic effect. Importantly, this effect was observed when using cells from two different MM cell lines, but also when using primary plasma cells isolated from the BM of MM patients.

### Thymoquinone decreases the surface expression of CXCR4 on MM cells and CXCL12-induced CXCR4/CD45 interactions

**Figure 5:**
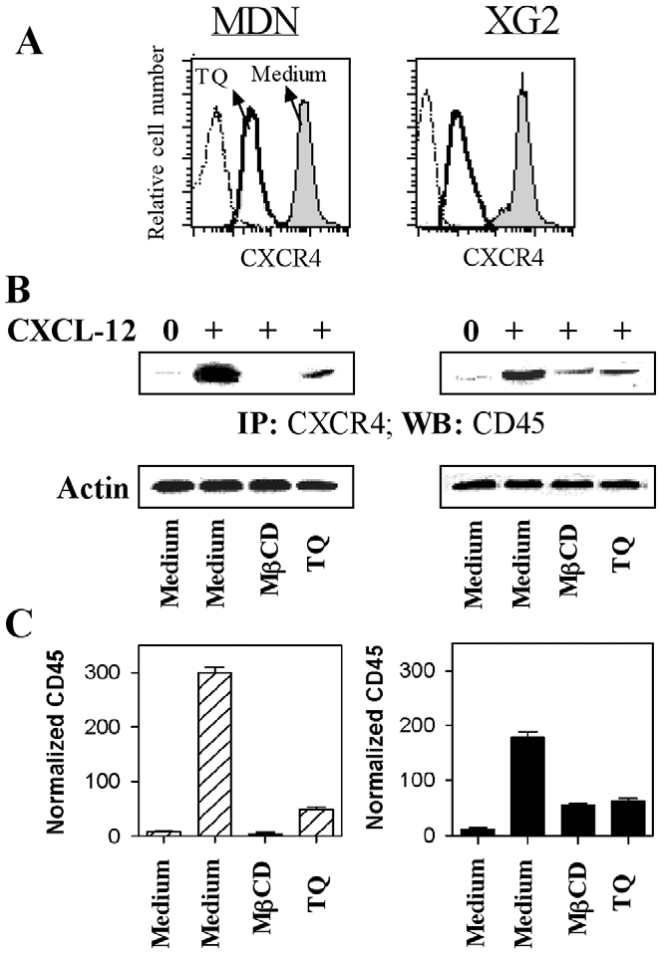
TQ decreases CXCR4 expression on MM cells and CXCL12-induced CXCR4/CD45 association. (**A**) MDN (left) and XG2 (right) cells were incubated for one hour at 37°C in medium with (bold solid line histograms) or without (gray filled histograms) 10 µM TQ, before being stained with an anti-CXCR4 mAb and analyzed by flow cytometry. Cells stained with an isotype-matched mAb are shown as a negative control (thin dotted line histograms). (**B**) MDN (left) and XG2 (right) cells were incubated with or without 2 mM MβCD for 30 min at 37°C or 10 µM TQ for one hour prior to incubation for 2 min in medium with (+) or without (0) 250 ng/ml CXCL12. The cells were then lysed and immunoprecipitated (*IP*) with a CXCR4 antibody. The immune complexes were separated on a 7% SDS-PAGE gel, transferred to a nitrocellulose membrane, and immunoblotted with an anti-CD45 antibody (upper blot). Equal protein loading was confirmed by running 50 µg of the total lysates on a SDS-PAGE gel and immunoblotting using an anti-actin antibody (lower blot). A representative blot from 6 independent experiments is shown. (**C**) Results from figure 5C are expressed as mean normalized CD45 levels ± SEM from six separate experiments.

We subsequently investigated the mechanisms involved in TQ-mediated inhibition of MM cell chemotaxis. For this purpose, we first assessed the CXCR4 expression levels in both MDN and XG2 cells before and after incubation with 10 µM TQ. In the absence of TQ, CXCR4 was strongly expressed by both MM cell types (mean fluorescence intensity, MFI = 521±5.2 and 448±7.1 in MDN and XG2 cells, respectively; n = 5). However, TQ strongly decreased CXCR4 surface expression of both MDN (MFI = 48±4.9; n = 5) and XG2 (MFI = 16±7.2; n = 5) cells (**Figure 5A**). We next investigated whether the CXCL12-mediated interaction of CD45 with CXCR4, which we showed was important for CXCL12-induced chemotaxis of MM cells, was also affected by TQ. To address this question, MDN and XG2 cells were cultured in medium with or without 10 µM TQ or 2 mM MβCD (which extracts cholesterol from the cell membrane and disrupts lipid rafts) prior to stimulation with CXCL12. We showed that the CXCL12-induced interaction of CXCR4 with CD45 was strongly impaired by pre-treatment with TQ (**Figure 5B**). However, this effect may result from the reduced expression of CXCR4 on the surface of MM cells rather than from a direct TQ effect on CXCR4/CD45 interaction. Finally, we showed that pre-treatment with MβCD completely abolished the CXCL12-induced association of CXCR4 with CD45 in both MDN and XG2 cells, suggesting that lipid rafts are also required for CXCL12-induced MM cell chemotaxis. Similarly to previous reports showing that MβCD treatment did not modulate the expression of surface receptors, such as B Cell Receptor, CD4 or CXCR4 [41], [42], we showed that MβCD treatment did not modulate the expression levels of CXCR4 or CD45 on the surface of MM cells (data not shown).

### TQ increases CD95 expression levels and susceptibility to Fas-mediated apoptosis of MM cells

**Figure 6:**
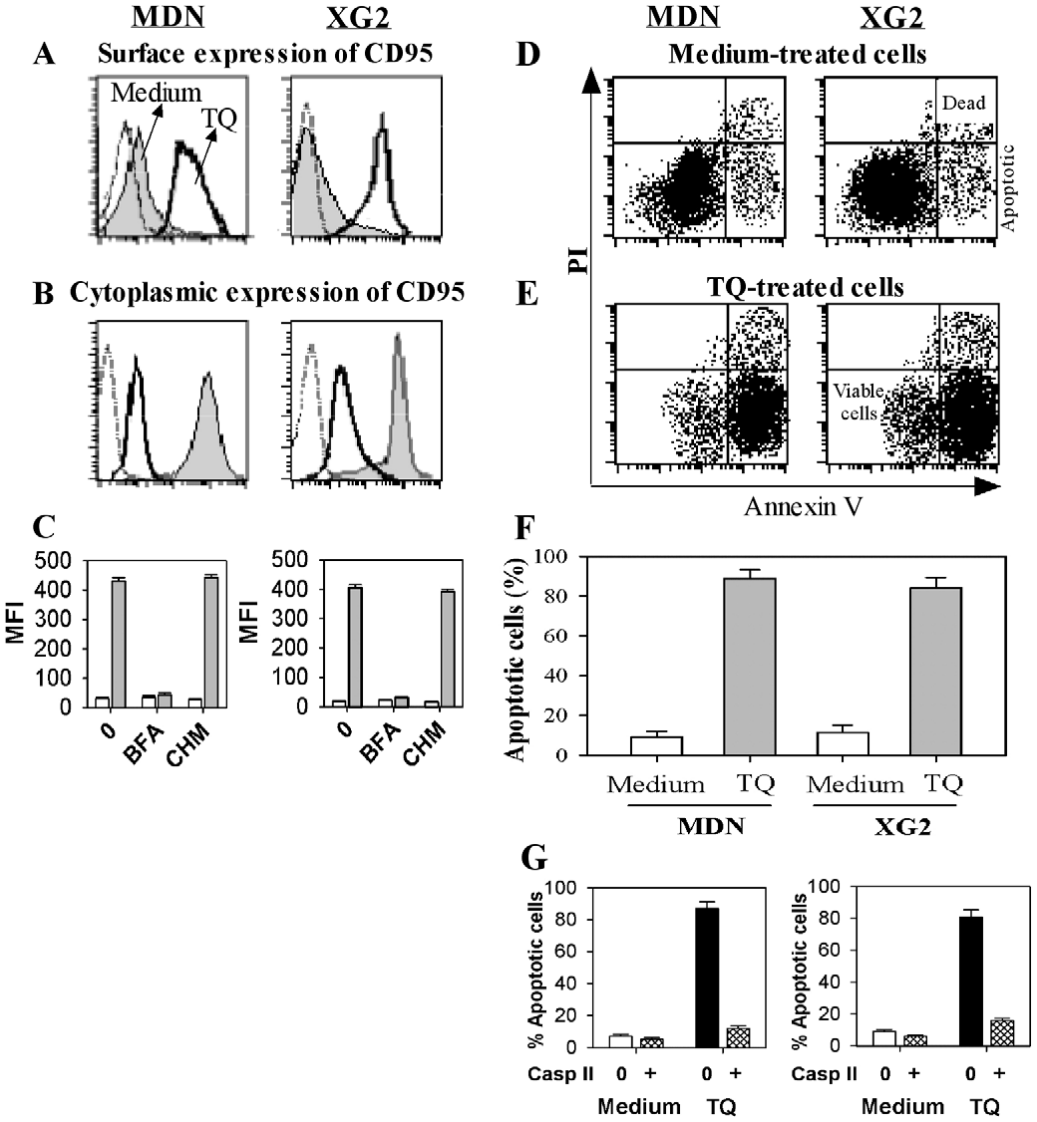
TQ increases the surface expression of CD95 and susceptibility of MM cells to Fas-mediated apoptosis. MDN and XG2 cells were incubated for one hour at 37°C in medium either with (bold solid line histograms) or without (gray filled histograms) 10 µM TQ prior to assessing the levels of extracellular (**A**) and intracellular (**B**) CD95 expression by flow cytometry. Cells were stained with an isotype-matched mAb are shown as a negative control (thin line histograms). One representative data set from eight independent experiments is shown. (**C**) MDN (left) and XG2 (right) cells were incubated for 30 min at 37°C with medium alone (0), 10 µg/ml brefeldin A (BFA) or 1 µM cycloheximide (CHM) prior to incubation for one hour with (gray bars) or without (open bars) 10 µM TQ. CD95 surface expression levels were assessed by flow cytometry and results are expressed as the mean fluorescence intensity (MFI) value ± SEM from nine separate experiments. (**D** & **E**) MDN and XG2 cells were incubated for one hour at 37°C in medium either with (E) or without (D) 10 µM TQ prior to starvation for 24 h in the presence of 1 µg/ml agonistic CD95 mAb (clone BG-27). The percentage of cells undergoing apoptosis was determined by flow cytometry based on the PI/Annexin V staining patterns. The percentage of apoptotic cells was consistently below 10% in all cells incubated for 24 h in the presence of a CD95-unrelated mouse isotype control IgG2a. One representative data set from nine independent experiments is shown. (**F**) Same as (D&E) but the results are expressed as the mean percentage of apoptotic cells ± SEM from nine separate experiments. (**G**) MDN (left panel) and XG2 (right panel) cells were incubated for one hour at 37°C in medium either with or without 10 µM TQ prior to a further incubation for one hour at 37°C with or without 10 µM Casp II. The cells were then starved for 24 h in the presence of 1 µg/ml B-G27 mAb, and the percentage of apoptotic cells was determined by flow cytometry. The results are expressed as the mean percentage of apoptotic cells ± SEM from nine separate experiments.

We assessed the effect of TQ pre-treatment on the surface expression of various markers that could be involved in the chemotaxis and viability of MM cells. TQ did not significantly modulate surface expression levels of LFA-1 (CD11a/CD18), ICAM1 (CD54), CD62L, CD5, CD10, CD80, CD86, CD23, CD38, CD44 or CD27 (data not shown). In contrast, the surface expression level of CD95 was strongly increased in TQ-treated MDN cells (MFI = 24±8.9 and 233±11 in untreated and TQ-treated cells, respectively; n = 8) and XG2 cells (MFI = 15±7 and 289±12 in untreated and TQ-treated cells, respectively; n = 8) (**Figure 6A**). The increased CD95 surface expression observed in TQ-treated cells was inversely correlated with a decrease in cytoplasmic CD95 expression (**Figure 6B**). Moreover, the TQ-mediated CD95 up-regulation was abolished by the addition of 10 µg/ml brefeldin A but not 1 µM cycloheximide (**Figure 6C**), suggesting that TQ exerts its effect by inducing the relocation of intracellular CD95 to the cell surface without inducing any *de novo* CD95 protein synthesis.

Since TQ treatment increased CD95 expression on MM cells, we investigated whether TQ could also increase their susceptibility to CD95-mediated apoptosis. For this purpose, both untreated and TQ-treated MDN and XG2 cells were incubated for 24 h in the presence of 1 µg/ml B-G27 mAb (agonistic CD95 mAb) prior to staining with Annexin-V and PI to discriminate among viable, necrotic/dead and apoptotic cells (**Figure 6D&E**). The percentage of apoptotic MDN cells increased from 9.3±2.7% to 88.7±4.6% (n = 9) when the cells were treated with TQ (**Figure 6F**). Similarly, the percentage of apoptotic XG2 cells increased from 11.4±3.7% to 84.1±5.2% (n = 9) when cells were treated with TQ (**Figure 6F**). In addition, we showed that the effect of TQ on Fas-mediated apoptosis was completely inhibited by pre-treatment of MM cells with a pan-caspase inhibitor (**Fig. 6G**).

## Discussion

Although the pro-apoptotic and cytostatic (growth arrest) effects of TQ on numerous cancer cells have been known for many years [25], [26], [27], [28], [29], [30], [31], very little is known concerning its effects on MM cells. Here, we investigated the effects of TQ on two different MM cell lines (MDN and XG2) as well as on primary plasma cells isolated from the BM of MM patients. Because chemotaxis is important and critical for the homing, interaction with the BM microenvironment and survival of MM cells, we first assessed: (i) the chemokine receptor expression patterns of MDN and XG2 cells; (ii) their migratory capacity toward various chemokines and (iii) the signaling pathways activated upon their stimulation with chemokines. We showed that MDN and XG2 cells strongly expressed CCR9, CXCR3 and CXCR4; expressed low levels of CCR1, CCR5, CCR7 and CXCR5 and did not express CCR6. Among these chemokine receptors, we showed that only CXCR4 was fully functional and enabled MDN and XG2 cells to specifically migrate in response to CXCL12. Our results are in agreement with reports showing that CXCL12, through activation of CXCR4, attracts human MM cells to the endothelial border, as well as cells from MM cell lines such as 5T33MM cells [43], [44].

The signaling pathways involved in CXCL-12-mediated MM cell chemotaxis are poorly defined and require further clarification. We therefore analyzed the effects of various inhibitors on the CXCL12-mediated MDN and XG2 cell chemotaxis. Our results provided strong evidence for the involvement of PLC, Class II PI3K/AKT/Rho-A, IκBα/NFκB and ERK, but not of Class I PI3K or P38 MAPK, in CXCL-12/CXCR4-mediated MM cell chemotaxis. This is in agreement with a previously published report that demonstrated (i) the involvement of PI3K and ERK but not P38 in CXCL-12-mediated MM cell chemotaxis and (ii) that AMD3100 significantly inhibits the homing of MM cells to the BM [20]. To better define and confirm the signaling pathways activated following the interaction of CXCL-12 with CXCR4 in MDN and XG2 cells, we performed western blot analyses. We found that CXCL12 specifically enhanced PLCβ3 and ERK1/2 phosphorylation as well as Rho-A activation, but had no effect on P38MAPK phosphorylation. Since previous studies of acute lymphoblastic leukemia have demonstrated that p38 MAPK is a critical regulator of cell migration [45], our findings highlight the differences in the signaling pathways activated in various malignant cell types.

Fernandis *et al.* showed that CXCL12 stimulation induces the association of CXCR4 with CD45 in the Jurkat T-cell line [40]. Since Na_3_VO_4_ inhibited the CXCL12-mediated chemotaxis of MDN and XG2 cells, we hypothesized that the membrane tyrosine phosphatase CD45 may be involved in CXCL12-mediated MM cell chemotaxis. Accordingly, we showed that CXCL12-mediated MM cell chemotaxis requires the association of CXCR4 with CD45. This is an important finding since CD45 is a critical regulator of signaling thresholds in immune cells [46] that regulates the retention, motility, and numbers of hematopoietic progenitors in the BM [47]. More importantly, we found that the CXCL12-induced association of CXCR4 with CD45 is strongly inhibited by pre-treating MM cells with TQ, and our results suggest that this inhibition is dependent on TQ-mediated down-regulation of CXCR4 expression. Nevertheless, such CXCR4 down-regulation likely represents an important TQ mechanism of action because we also showed that TQ decreases CXCL12-mediated MM cell chemotaxis in a dose- and time-dependent manner. Such TQ-mediated CXCR4 down-regulation could be particularly relevant for clinicians, in light of the report by Alsayed et al. showing that disease activity is inversely correlated with CXCR4 expression levels on MM cells [20]. Finally, it is worth noting that TQ inhibited CXCL12-mediated chemotaxis in MM cells but not in PBMCs isolated from healthy individuals. Furthermore, TQ did not affect cell viability, highlighting the fact that TQ exerts a potent and specific effect on MM cells without inducing any cytotoxic effect.

Another major finding emerging from the present study is the demonstration that the surface expression of CD95 is strongly increased on TQ-treated MM cells. We showed that this up-regulation results from the relocation of intracellular CD95 to the cell membrane and not from *de novo* protein synthesis. Such relocation of intracellular CD95 to the cell surface has previously been observed in other cell types, e.g. in untransformed human vascular smooth muscle cells and pancreatic β-cells following p53 activation and/or their stimulation with interferon gamma [48], [49], [50]. In particular, Bennett et al. specifically showed that the intracellular CD95 was localized to the Golgi complex and trans-Golgi network, and its relocation to the cell membrane did not require protein synthesis [48]. It is however worth mentioning that the requirement of protein and RNA synthesis for the translocation of CD95 to the cell surface appears to be dependent on the cell type under investigation [50]. CD95 plays a major role in apoptosis. Indeed, stimulating CD95 via an agonistic anti-CD95 antibody or its ligand (CD95L) results in CD95 oligomerization and formation of the death-inducing signaling complex (DISC), which in turn activates a series of caspases and results in apoptotic cell death [51]. Therefore, we investigated whether TQ-mediated CD95 up-regulation was functionally significant and increased MM cell susceptibility to apoptosis. Our results showed that TQ treatment increases CD95-mediated, caspase-dependent apoptosis of MM cells by up to 85%. To our knowledge, we show here for the first time that TQ up-regulates CD95 expression on the surface of MM cells and increases their susceptibility to Fas-mediated apoptosis. Interestingly, Gali-Muhtasib *et al.* previously reported a pro-apoptotic activity of TQ treatment, though in a different cancer model (colorectal cancer cells) and involving a different mechanism of action (triggering the inactivation of the stress response pathway sensor CHEK1) [52]. Recently, Li et al. also reported that TQ inhibits the proliferation and induces the apoptosis of MM cells through the suppression of stat3 activation pathway [53]. In conclusion, the present data expand our knowledge of TQ mechanism of action and suggest that TQ may represent a promising drug candidate for the treatment of patients with MM, providing a rationale for its clinical evaluation.

## Supporting Information

Figure S1 **Chemokine receptor expression by MM cells.** The chemokine receptor expression levels (gray filled histograms) of XG2 cells were determined by flow cytometry using specific monoclonal antibodies (mAbs). Cells stained with an isotype-matched mAb are shown as a negative control (thin solid line histograms). One representative data set from four independent experiments is shown.
(TIF)
