# Supplementary figures and images for: Thymoquinone Inhibits the CXCL12-Induced Chemotaxis of Multiple Myeloma Cells and Increases Their Susceptibility to Fas-Mediated Apoptosis
Source: PLoS One. 2011 Sep 1;6(9):e23741. doi: 10.1371/journal.pone.0023741 (PMC3164673; doi:10.1371/journal.pone.0023741)

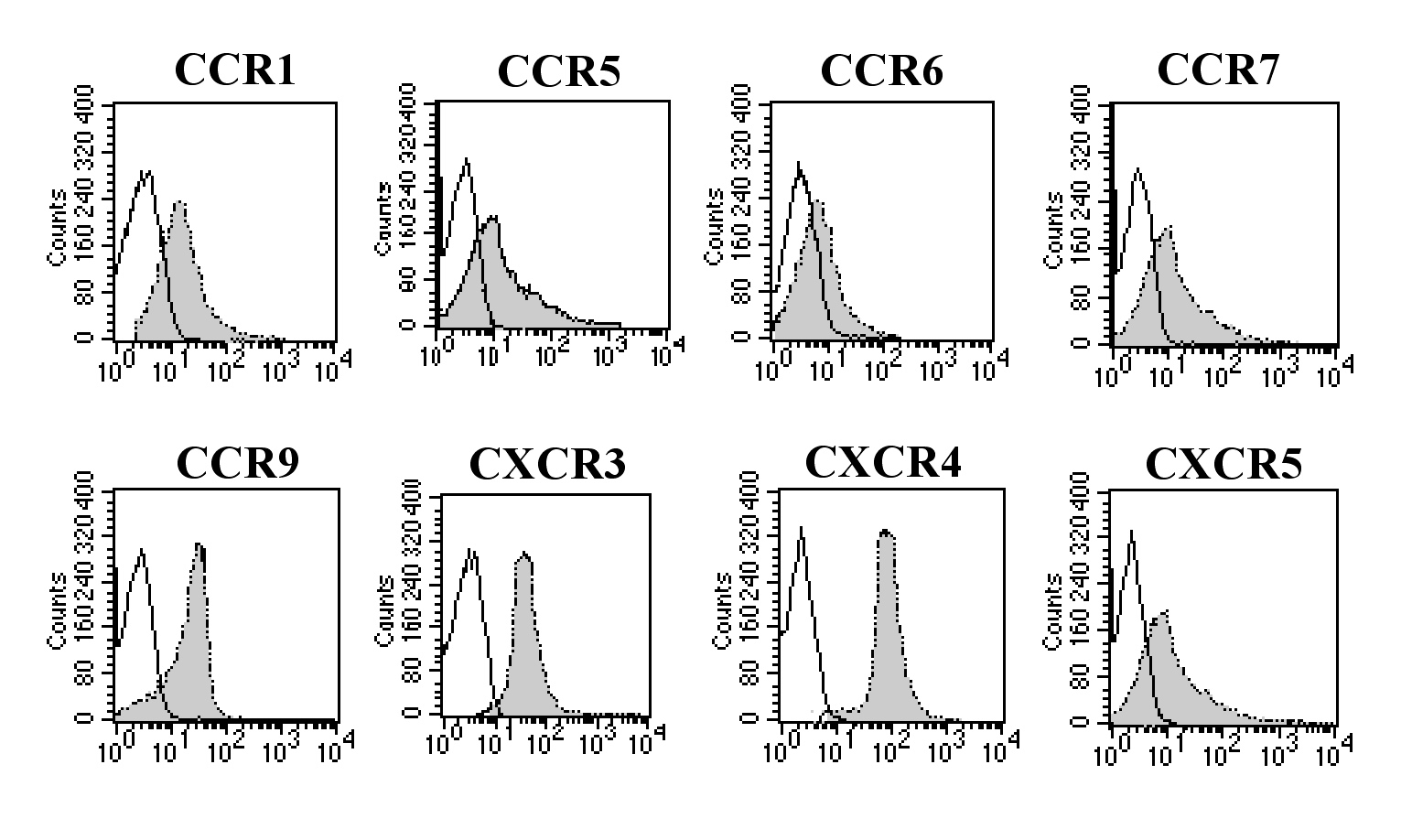

Supplement: Figure S1 — Chemokine receptor expression by MM cells. The chemokine receptor expression levels (gray filled histograms) of XG2 cells were determined by flow cytometry using specific monoclonal antibodies (mAbs). Cells stained with an isotype-matched mAb are shown as a negative control (thin solid line histograms). One representative data set from four independent experiments is shown. (TIF) [file pone.0023741.s001.tif]
